# Neonatal mortality in small for gestational age infants based on reference local newborn curve at secondary and tertiary hospitals in Indonesia

**DOI:** 10.1186/s12887-023-04023-z

**Published:** 2023-05-05

**Authors:** Ekawaty L. Haksari, Mohammad Hakimi, Djauhar Ismail

**Affiliations:** 1 Department of Child Health, Faculty of Medicine, Public Health and Nursing, Universitas Gadjah Mada, Sardjito General Hospital, Yogyakarta, Indonesia; 2 Department of Obstetrics & Gynaecology, Faculty of Medicine, Public Health and Nursing, Universitas Gadjah Mada, Yogyakarta, Indonesia

**Keywords:** Neonatal mortality, Small for gestational age, Preterm infants, Reference curve

## Abstract

**Background:**

Small for gestational (SGA) infants during the neonatal period have risks of mortality and sequelae for survival. Two - third of neonatal mortality occurs in the first weeks of life. Prevalence of SGA depends on the newbon curve used. Objectives of the study were to know the conditions that posed the risk of early neonatal and neonatal mortality, to identify preterm/full-term and SGA/appropriate gestational age (AGA) infants with cumulative mortality incident (CMI), to compare 5- year-period of early and neonatal mortality, and to investigate CMI on neonatal mortality of four categories during 5-year-period.

**Methods:**

A retrospective cohort study on all live births, during 1998–2017, was conducted in Sleman and Sardjito hospitals, Yogyakarta, Indonesia. Based on the reference local curve, the eligible subjects were categorized into SGA and AGA infants. The analyses were based on preterm/full-term and SGA/AGA, thus resulting in 4 categories: preterm-SGA, preterm-AGA, full-term-SGA and full-term-AGA. Analysis was made with Unadjusted Hazard Ratio (HR) by Simple Cox Regression and Adjusted HR was calculated by Multiple Cox Regression, survival analysis to calculate CMI, and analysis mortality for 5-year period ( 1998–2002, 2003–2007, 2008–2012, 2013–2017).

**Result:**

There were 35,649 live births eligible for the study. Respiratory distress was the highest risk with HR 9,46, followed by asphyxia with HR 5,08, mother’s death with HR 227, extra-health facility with HR 1,97, symmetrical SGA with HR 1,97, preterm-AGA with HR 1,75, low birth weight (LBW) with HR 1,64, primary health facility with HR 1,33, and boys with HR 1,16 consecutively. Early neonatal mortality in 4 categories by survival analysis revealed the highest CMI in preterm SGA. Similar result was found in neonatal mortality. Analysis of 5-year period unveiled the highest CMI during 1998–2002. The highest CMI based on the four categories, however, was found in preterm-SGA.

**Conclusion:**

Respiratory distress posed the highest HR in early and neonatal mortality. Survival analysis showing the highest CMI on early and neonatal mortality was identified in preterm-SGA. The 5 - year - period of neonatal mortality showed the highest CMI during 1998–2002 period, whereas based on 4 SGA categories, preterm-SGA demonstrated the highest CMI.

## Introduction

Indonesia is one of 11 countries with over 15% preterm, and one of 10 countries with the highest number of small for gestational age (SGA) infants in the world [1, 2]. There were 51% SGA and 41% premature low birth weight (LBW) infants [3].

In developing countries, with high prevalence of LBW, there are SGA infants with various levels [2, 4]. The prevalence of SGA in Nepal, for instance varies from 10,5 to 72,5% and in India from 12 to 78,4%. Boys SGA outnumbered girls. When local curve is used or based on low-middle countries, the prevalence of SGA will decrease [4]. In this way, an appropriate curve is required to evaluate the prevalence of SGA that could be used as a guide to facilitate examinations [5–7].

Neonatal period, especially at birth, and early neonatal period, are a vulnerable and complicated phases. Two-third neonatal mortality occurs in the first weeks of life [8]. Premature babies are generally at high risk during neonatal and post-neonatal periods. Small for gestational age infants, on the other hand, have higher neonatal risk mortality than full term and preterm AGA infants [9]. SGA infants have 29% neonatal mortality and 26% infant mortality [10]. The most common risks of neonatal mortality are prematurity and SGA [9–12].

Problems of SGA infants in neonatal period lead to mortality and sequelae for survival. Additionally, SGA infants are fragile individuals who require greater health care, especially in low-and middle-income countries [2, 9, 14].

Some efforts to reduce neonatal mortality are necessary since it contains 45% of under five -year- mortality, which is difficult to decrease [15]. Indonesia has reportedly had the highest neonatal mortality [16], with approximately 59% infant mortality occurring during the neonatal period. Indonesia Survey of Demography and Health 2017 reported neonatal mortality of 15/ 1000 live births [17, 18].

Clinicians in the countries without a newborn curve based on their population will use the available curve. Recently, plenty of developing countries including Indonesia have used Lubchencho’s curve. Reference neonatal curves for birthweight, supine length, and head circumference have been developed in Yogyakarta, Indonesia, to classify high risk newborns and identify newborns requiring attention [19].

The objectives of this study were to know the conditions that posed the risk of early neonatal and neonatal mortality, to identify preterm/full-term and SGA/appropriate gestational age (AGA) infants with cumulative mortality incident (CMI), to compare 5- year-period of early and neonatal mortality, and to investigate CMI on neonatal mortality of four categories during 5-year-period.

**Fig. 1:**
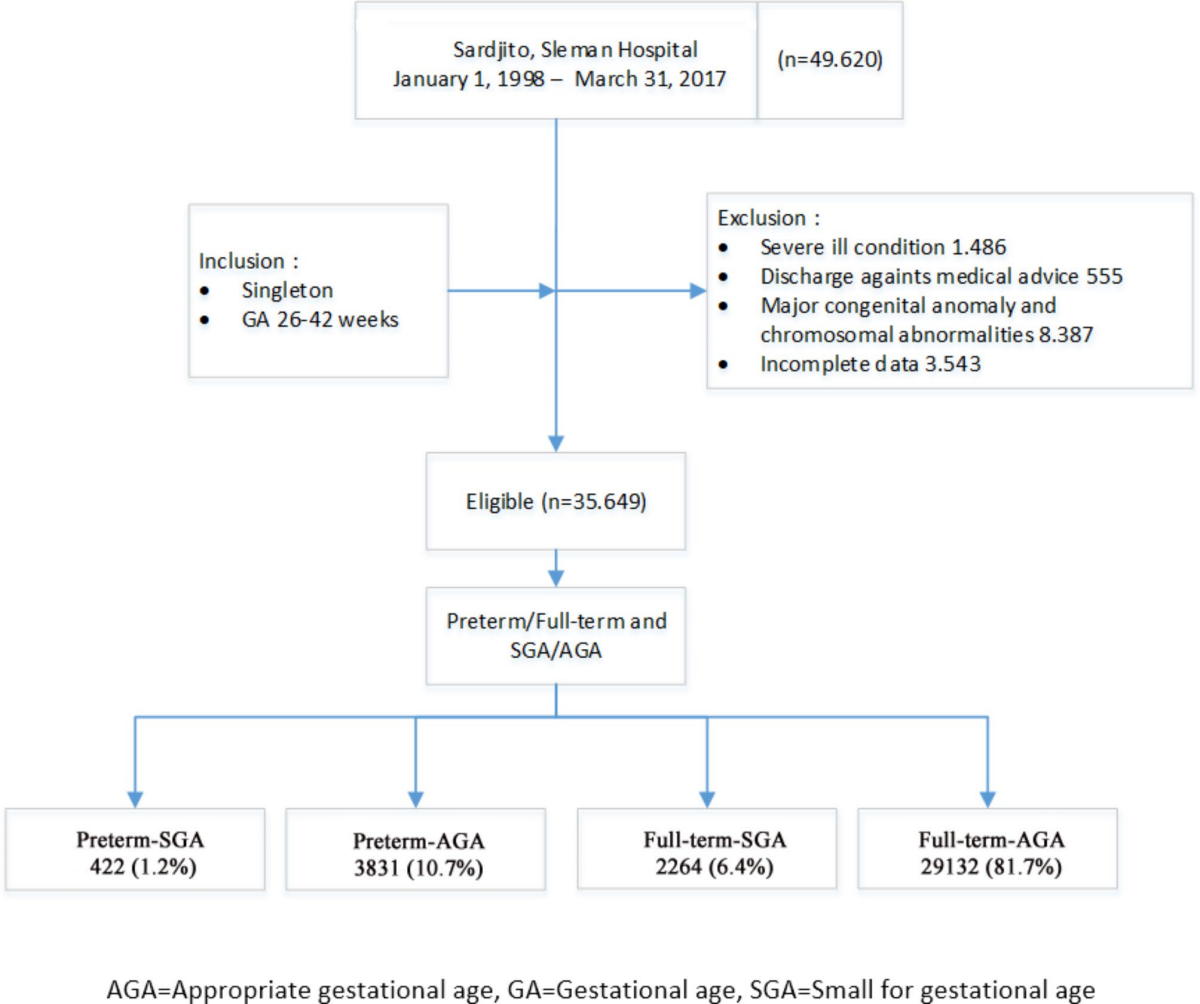
The course of the study

**Table 1:** Basic characteristics of newborns

	Total
N	35,649
Sex	
Boy	18,933 (53.1%)
Girl	16,716 (46.9%)
Birth weight	
>=2500 g	29,154 (81.8%)
2000–2499	3,913 (11.0%)
1500–1999	1,540 (4.3%)
1000–1499	795 (2.2%)
<=999	247 (0.7%)
Gestational age	
<28 weeks	285 (0.8%)
28–32	898 (2.5%)
33–34	1,072 (3.0%)
35–36	1,998 (5.6%)
37+	31,396 (88.1%)
Nutritional status	
Symmetrical	719 (2.0%)
Asymmetrical	3,245 (9.1%)
Normal	31,685 (88.9%)
Asphyxia	
Yes	4,162 (11.7%)
No	31,487 (88.3%)
Respiratory Distress	
Yes	2,860 (8.0%)
No	32,789 (92.0%)
Sepsis	
Yes	3,052 (8.6%)
No	32,597 (91.4%)
Birth Trauma	
Yes	213 (0.6%)
No	35,436 (99.4%)
Mode of delivery	
Spontaneous	30,696 (86.1%)
Vaginal assistance	816 (2.3%)
Caesarean section	4,137 (11.6%)
Place of birth	
Tertiary hospital	16,883 (47.4%)
Secondary hospital	17,444 (48.9%)
Primary facility	1,046 (2.9%)
Others	276 (0.8%)
Neonatal mortality	
Early neonatal	1,100 (3.1%)
Late neonatal	287 (0.9%)
Live	34,082(96.0%)

## Materials and methods

### Study population and study period

This study was the second phase of the study on mortality and morbidity in small for gestational age infants [20].

Primary health care services in Indonesia are provided in health centres and private clinics. District hospitals are secondary health facilities that provide referral services in the area. Meanwhile, tertiary health facilities are made available at teaching hospitals, which are commonly found in the capital of a province. However, in provinces without teaching hospitals, the services are provided by the provincial hospitals.

**Fig. 2:**
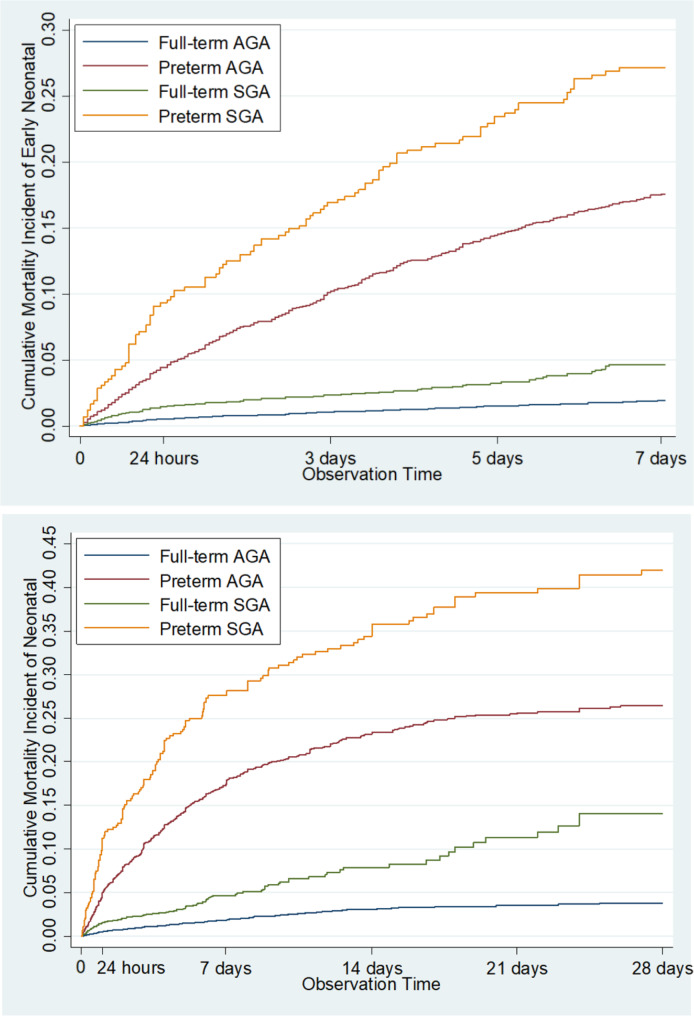
Cumulative mortality incident of early neonatal and neonatal mortality on preterm-SGA, preterm-AGA, full-term SGA, and full-term SGA

Cohort retrospective design was used for live births at Sardjito-a tertiary hospital, and Sleman district hospital- a secondary hospital in Yogyakarta; from January 1, 1998 to March 31, 2017.

Total sampling was conducted to all live birth newborns cared at Sardjito and Sleman hospitals from January 1, 1998 to March 31, 2017. Meanwhile, the sample size was 2741 [21]. Eligible samples were based on the inclusion and exclusion criteria.

### Study variable and operational definition

The dependent variable was early neonatal and neonatal mortality during hospital stay. Neonatal mortality was divided into early and late neonatal mortality. It refers to the mortality during the neonatal period, any death that occurred during 0 to 28 days after birth. Early neonatal mortality was defined as any death that occurred from 0 to 7 days. The independent variable was SGA, which was based on the reference local newborn curve (19). The infants with less than 10 percentiles by gestational were categorized into SGA, while those with more or equal to 10 percentiles were classified as AGA. Gestational age was estimated in weeks by using Dubowitz score or, if not possible, by Ballard score [22, 23]. Dubowitz score was used to establish the clinical assessment of gestational age for newborns, while Ballard score was applied to very preterm and sick neonates.

The confounding variable consisted of the group of newborn, mother, and others. The variables of the newborns were sex, low birth weight (LBW), nutritional status, asphyxia, respiratory distress, sepsis, and birth trauma. Meanwhile, mothers’ variables were mother’s age, mother’s education, parity, ante natal care (ANC), and maternal mortality. The other variables were mode of delivery and place of birth.

Sex referred to boy and girl physically. The newborns were weighed soon after birth and were consistently reported in the medical records (gram) until they were classified into < 2500 gram (LBW) and > 2500 gram. Preterm was defined gestational age < 37 weeks, while full-term was with gestational age ≥ 37 weeks.

Nutritional status referred to the SGA infants, both symmetrical and asymmetrical. Symmetrical means both birth weight and body length were < 10 percentile compared with gestational age. On the contrary, if birth weight was < 10 percentile but body length was > 10 percentile compared with gestational age, it was considered asymmetrical. Those who did not belong to SGA were put into the normal category. Asphyxia was defined as no breathing at birth. Apgar score in 5 min was < 6, or 2 cycles positive pressure ventilation was needed, or PH < 7.2 of blood gas analysis was necessary. The respiratory distress was clinically assessed with Downes score [24–26] and diagnostic support. Sepsis was confirmed clinically when there was at least 1 case in 4 of 6 groups (general condition, cardiovascular, gastrointestinal, respiration, central nervous system, haematology) or there was a positive bacterial culture in the body fluid, by clinical practice guidelines of Sardjito. Birth trauma referred to a condition when trauma was detected or there was a delivery-related injury in the newborns, which was subsequently classified into Yes and No.

Mother’s age referred to the age of mother when she gave birth and was classified into < 18, 18–35 and > 35 years. Mother’s education was consistent with the report recorded in the medical records, which was categorized into ≤ 6 years, 7–12 years and > 12 years. Parity was defined as the number of mothers giving birth as consistently reported in the medical records. They were classified into 1, 2–4, and > 5. Antenatal care (ANC) was defined as the number of examinations undergone by mothers during pregnancy, which were subsequently reported in the medical records and were classified as 0, 1–4, and > 5. Maternal mortality is a condition when a mother died as being discharged from the hospital, which subsequently was classified into Yes or No.

The delivery methods included spontaneous delivery, assisted spontaneous delivery, and caesarean section. Birth place was defined as the venue where the infant was born, which was then assorted to Tertiary hospital, Secondary hospital, Primary health facilities, and Extra health facilities.

The inclusion criteria were singleton and gestational age 26–42 weeks. Meanwhile, the exclusion criteria included major congenital anomalies, chromosomal abnormalities, severe ill conditions, discharge against medical advice, and incomplete data.

### Data collection

During the study, the data were recorded as soon as the baby was born. Re-check and re-examination were done to match all of the data. Trained administrators would entry the data into the database.

The data were collected from The Maternal-Perinatal (MP) database, South East Asia Region Neonatal-Perinatal Data (SEAR NPD WHO) forms, and medical records if the data were incomplete. The data collected at the Sleman hospital were from MP database, whereas the ones collected at Sardjito hospital during January 1,1998-December 31,2007 were from MP data base and during January 1, 2008- 31 March,2017 were from SEAR NPD WHO forms. The MP database was part of the MP audit, which was a district-based audit of maternal, perinatal, and neonatal mortality. SEAR NPD WHO forms concerning maternal and neonatal perinatal database were issued by WHO, which were initially applied in India. It was subsequently implemented in Bangladesh, Sri Lanka, Thailand, and Indonesia at the end of 2007.

### Data analysis

The study was investigating early neonatal mortality and neonatal mortality. Based on the reference local curve, the eligible subjects were categorized into SGA and appropriate gestational age (AGA) infants. The analyses were based on preterm/full-term and SGA/AGA, thus resulting in 4 categories: preterm-SGA, preterm-AGA, full-term-SGA, and full-term-AGA.

The analyzed variables were the ones with early neonatal and neonatal mortality risks. If Unadjusted Hazard Ratio (HR), which was calculated by Simple Cox Regression, unveiled a significant difference; it would subsequently be analyzed using Adjusted HR calculated by Multiple Cox Regression.

Survival analysis was conducted on early neonatal and neonatal mortality. In our study, observation started from birth, 24 h, 3 days, until 7 days for early neonatal death, and until 28 days for neonatal mortality. The survival analysis curve was used to compare the CMI of early and neonatal mortality during hospitalization.

The study was carried out for 20 years (1998–2017). Analysis on early neonatal and neonatal mortality was compared for 5-year-period (1998–2002, 2003–2007, 2008–2013, 2014–2017. Then CMI of neonatal in preterm-SGA, preterm-AGA, Full-term-SGA dan Full-term-AGA was calculated. Additionally, the four groups were compared for 5-year-period (1998–2002, 2003–2007, 2008–2013, 2014–2017).

The power of the study was calculated by the sample which met the criteria and that could be analysed at the end of the study. All stages of analyses were performed using Stata 13 software.

## Results

Of 49,620 live birth newborns from Sleman district hospital and Sardjito hospital, 35,649 were eligible for the study (Fig. 1).

Preterm infants constituted 11,9% of the total newborns, with 7,2% in Sleman hospital and 16,5% in Sardjito hospital. Early neonatal mortality was 31/1000 live births and neonatal mortality was 40/1000 live births (Table 1).

There were 15.9% mothers aged < 18 years and > 35 years. Additionally, there were 80.5% mothers with parity 1 and ≥ 5. One hundred and sixty-two (0.5%) mothers died, 0.7% of whom passed away at Sardjito, which was higher than at Sleman hospital (0.2%) (Table 2).

**Table 2:** Basic characteristics of maternal

	Total
N	35,649
Mother’s age (y)	
<18	356 (1.0%)
18–35	29,980 (84.1%)
>35	5,313 (14.9%)
Mother’s education (y)	
≤6	4,555 (12.8%)
7–12	21,422 (60.1%)
≥13	9,672 (27.1%)
Parity	
1	28,454 (79.8%)
2–4	6,952 (19.5%)
≥5	243 (0.7%)
ANC	
0	6,726 (18.9%)
1–4	1,924 (5.4%)
≥5	26,999 (75.7%)
Maternal mortality	
Yes	162 (0.5%)
No	35,487 (99.5%)

ANC = ante natal care, y = year

Number of early neonatal mortality was 1,100. Early neonatal mortality had some previously significant variables by unadjusted HR analysis, i.e. preterm-SGA, full-term-SGA, maternal education, parity ≥ 5, asymmetrical SGA, sepsis, birth trauma, and non ANC. As they were put into adjusted HR, however, they turned out to be insignificant. Adjusted HR analysis unveiled respiratory distress was the highest risk with HR 9,46 (7,34 − 12,2), followed by asphyxia with HR 5,08 (4,09 − 6,32), maternal mortality with HR 2,27 (1,70 − 3,05), extra-health facility with HR 1,97 (1,44 − 2,69), symmetrical SGA with HR 1,97 (1.09–3.54), preterm-AGA with HR 1.75 (1.26–2.43), LBW with HR 1,64 (1,19 − 2,27), primary health facility with HR 1,33 (1,12 − 1,57) and boys with HR 1,16 (1,02 − 1,31) consecutively (Table 3). Similar result was found in neonatal mortality, for which the number of mortality was 1,387 (Table 4).

**Fig. 3:**
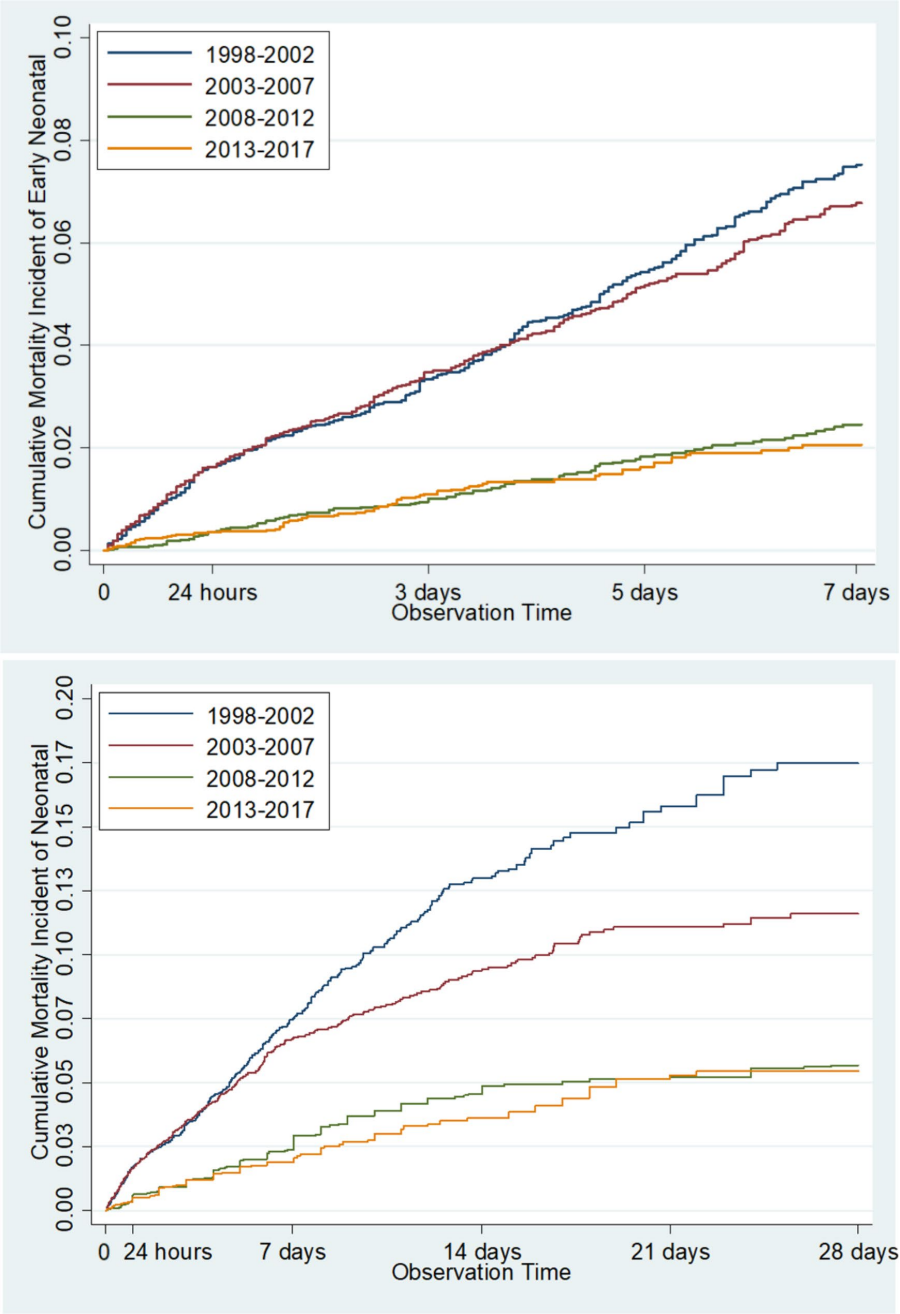
Cumulative mortality incident of early neonatal and neonatal mortality during 5 year- period

**Table 3:** Unadjusted HR and adjusted HR analyses on early neonatal mortality

	n (mortality)	Unadjusted	Adjusted
		HR [95% CI]	HR [95% CI]
Preterm/Full-term and SGA/AGA	1,100		
Full-term AGA	340	1[1]	1[1]
Preterm AGA	574	10,1***[8,78,11,5]	1,75***[1, 2, 26, 43]
Full-term SGA	76	2.39***[1,86,3,06]	0,61[0,33,1,13]
Preterm SGA	110	16.7***[5, 8, 13, 20]	1,35[0,74,2,47]
Mother’s age (y)	1,100		
< 18	16	1,27[0,77,2,08]	
18–35	917	1[1]	
> 35	167	0,98[0,83,1,16]	
Mother’s education (y)	1,100		
≤ 6	198	1,83***[1,52,2,21]	1,05[0,85,1,28]
7–12	651	1,36***[1,17,1,57]	1[0,85,1,18]
≥ 13	251	1[1]	1[1]
Parity	1,100		
1	872	0,99[0,85,1,15]	1,16[0,99,1,35]
2–4	214	1[1]	1[1]
≥ 5	14	1,94*[1, 3, 13, 33]	1,05[0.56,1,95]
Mode of delivery	1,100		
Spontaneous	995	1[1]	1[1]
Assisted vaginal delivery	19	0,72[0,46,1,13]	1,12[0,69,1,82]
Caesarean section	86	0,59***[0,47,0,73]	0,89[0,69,1,14]
Sex	1,100		
Boy	660	1,30***[1, 15, 47]	1,16*[1,02,1,31]
Girl	440	1[1]	1[1]
Birth weight (g)	1,100		
< 2500	756	7,71***[6.78,8.76]	1,64**[1, 2, 19, 27]
>=2500	344	1[1]	1[1]
Nutritional status	1,100		
Symmetrical	46	1,86***[1,38,2,50]	1,97*[1,09,3,54]
Asymmetrical	158	1,56***[1,31,1,85]	1,38[0,86,2,22]
Normal	896	1[1]	1[1]
Asphyxia	1,100		
Yes	889	27,7***[2, 7, 23, 32]	5,08***[4,09,6,32]
No	211	1[1]	1[1]
Respiratory distress	1,100		
Yes	915	48,0***[40,9,56.3]	9,46***[2, 7, 12, 34]
No	185	1[1]	1[1]
Sepsis	1,100		
Yes	426	4,55***[4,03,5,14]	0,81**[0,71,0,93]
No	674	1[1]	1[1]
Birth trauma	1,100		
Yes	19	2,39***[1,51,3,79]	1,09[0,68,1,74]
No	1,081	1[1]	1[1]
Antenatal care	1,100		
0	477	3,51***[3,10,3,98]	1,07[0,93,1,24]
1–4	112	3.08***[2,51,3,79]	1,15[0,92,1,44]
≥ 5	511	1[1]	1[1]
Maternal mortality	1,100		
Yes	71	15.7***[1, 3, 12, 20]	2,27***[1,70,3,05]
No	1,029	1[1]	1[1]
Birth place	1,100		
Tertiary hospital	729	1[1]	1[1]
Secondary hospital	122	0,21***[0,17,0,25]	0,52***[0,41,0,64]
Primary health facility	203	3,86***[3,29,4,52]	1,33***[1,12,1,57]
Extra health facility	46	4,00***[2,96,5,41]	1,97***[1,44,2,69]
Observations (n)		35,649	35,649

AGA = appropriate for gestational age, g = gram, HR = hazard ratio, LBW = low birth weight ,SGA = small for gestational age, y = year, *p < 0.05, **p < 0.01, ***p < 0.001

**Table 4:** Unadjusted HR and Adjusted HR analyses on neonatal mortality

	n (mortality)	Unadjusted	Adjusted
		HR [95% CI]	HR [95% CI]
Preterm/Full-term and SGA/AGA	1,387		
Full-term AGA	412	1[1]	1[1]
Preterm AGA	720	9,18***[4, 8, 10, 11]	1,71***[1, 2, 25, 34]
Full-term SGA	105	2,69***[2, 3, 17, 33]	0,66[0,38,1,17]
Preterm SGA	150	15,3***[5, 6, 12, 18]	1,41[0,80,2,49]
Mother’s age (y)	1,387		
< 18	19	1,02[0,64,1,61]	
18–35	1,147	1[1]	
> 35	221	1,07[0,92,1,23]	
Mother’s education (y)	1,387		
≤6	256	1,89***[1,60,2,22]	1,16[0,97,1,39]
7–12	788	1,37***[1,20,1,55]	0,99[0,85,1,14]
13+	343	1[1]	1[1]
Parity	1,387		
1	1,093	0,97[0,85,1,11]	1,17*[1,01,1,35]
2–4	276	1[1]	1[1]
≥ 5	18	1,97**[1, 3, 17, 22]	0,99[0,61,1,60]
Mode of delivery	1,387		
Spontaneous	1,225	1[1]	1[1]
Assisted vaginal delivery	26	0,82[0,56,1,21]	1,24[0,81,1,91]
Caesarean section	136	0,77**[0,65,0,92]	0,95[0,77,1,17]
Sex	1,387		
Boy	814	1,23***[1, 11, 37]	1,12*[1,00,1,26]
Girl	573	1[1]	1[1]
Birth weight (g)	1,387		
< 2500	972	7.45***[6,63,8,37]	1,77***[1, 2, 30, 42]
≥ 2500	415	1[1]	1[1]
Nutritional status	1,387		
Symmetrical	66	2,06***[1,61,2,63]	1,87*[1,09,3,21]
Asymmetrical	210	1,66***[1,43,1,93]	1,35[0,87,2,11]
Normal	1,111	1[1]	1[1]
Asphyxia	1,387		
Yes	1,050	19,5***[1, 2, 17, 22]	4,32***[3,63,5,14]
No	337	1[1]	1[1]
Respiratory distress	1,387		
Yes	1,101	33,6***[3, 4, 29, 38]	6,89***[5,63,8,43]
No	286	1[1]	1[1]
Sepsis	1,387		
Yes	644	5,33***[4,80,5,92]	1,09[0,96,1,23]
No	743	1[1]	1[1]
Birth trauma	1,387		
Yes	24	2,29***[1,52,3,44]	1,19[0,79,1,81]
No	1,363	1[1]	1[1]
Antenatal care	1,387		
0	585	3,36***[3,01,3,75]	1,12[0,98,1,28]
1–4	133	2,87***[2, 3, 38, 46]	1,07[0,87,1,32]
> 5	669	1[1]	1[1]
Maternal mortality	1,387		
Yes	89	14,8***[6, 8, 11, 18]	2,46***[1,91,3,17]
No	1,298	1[1]	1[1]
Birth place	1,387		
Tertiary hospital	939	1[1]	1[1]
Secondary hospital	147	0,22***[0,18,0,26]	0,52***[0,43,0,63]
Primary health facility	244	3,28***[2,83,3,79]	1,31***[1,12,1,53]
Extra health facility	57	3,77***[2,87,4,95]	2,04***[1,54,2,70]
Observations (n)		35,649	35,649

AGA = appropriate gestational age, CI = confident interval, g = gram, HR = hazard ratio, SGA = small for gestational age, y = year, *p < 0.05, **p < 0.01, ***p < 0.001

**Fig. 4:**
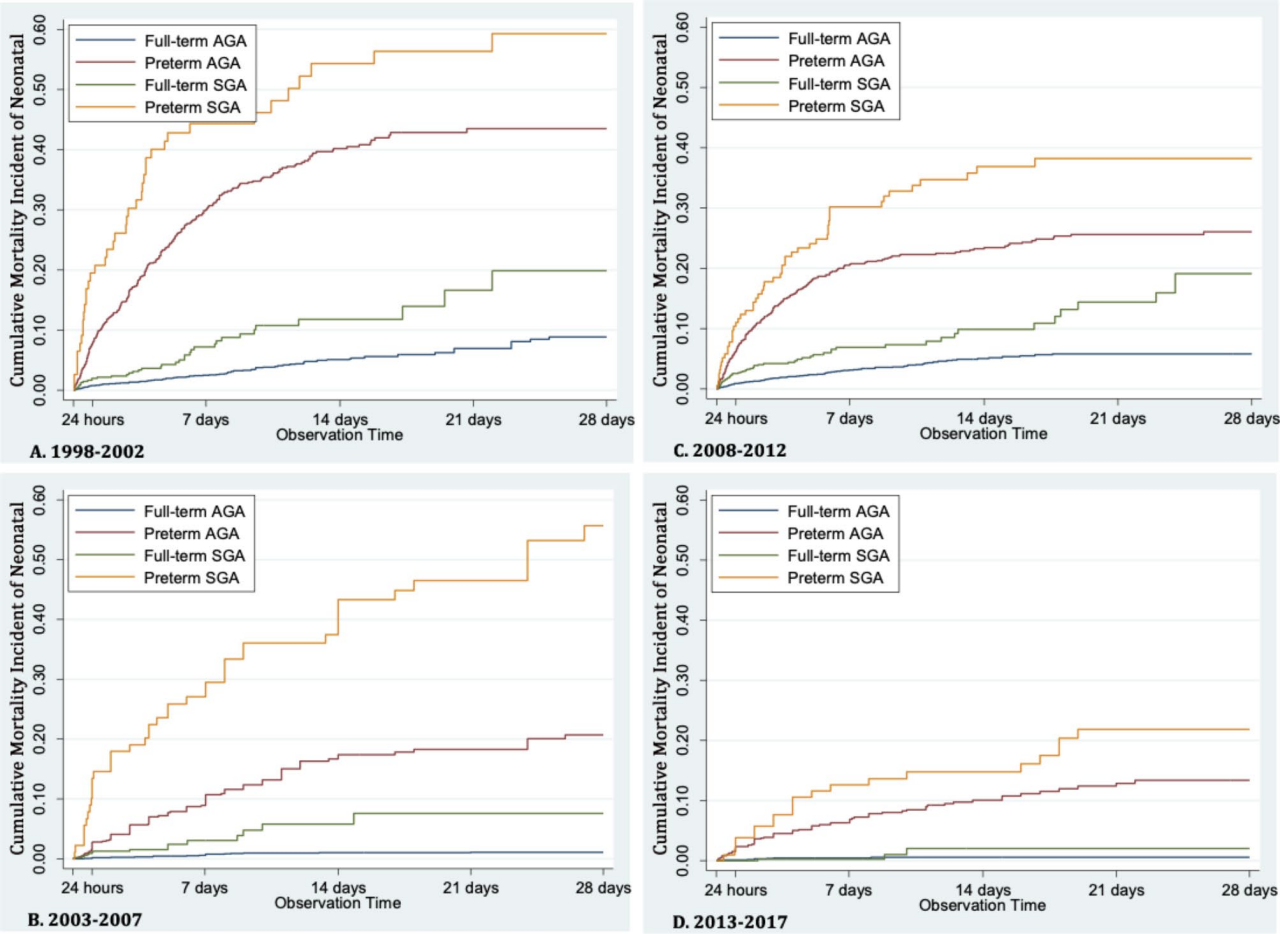
Neonatal mortality based on preterm-SGA, preterm-AGA, full-term-SGA, full-term- AGA during 5 year-period 1998–2017

Early neonatal mortality on survival analysis, with classification based on preterm/full-term and SGA/AGA showed the highest CMI was found in preterm-SGA, then in preterm-SGA and full-term-SGA consecutively, while the lowest was found in full-term-AGA. The result of neonatal mortality analysis was similar to that of early neonatal mortality (Fig. 2).

Survival analysis on the 5 - year - period of early neonatal mortality revealed that the highest CMI was recorded during 1998–2002, 2002–2007, and 2008–2012 successively, whereas the lowest CMI was found during 2013–2017. Similar result was reported in the neonatal mortality ( Fig. 3).

Survival analysis on neonatal mortality, with classifications based on preterm/full-term and SGA/AGA, during 5 - year - period ( 1998–2002, 2003–2007, 2008–2012, 2013–2017) displayed the highest CMI in preterm-SGA, preterm-AGA, and full-term-SGA consecutively, whereas the lowest CMI was noted in full-term-AGA (Fig. 4).

The power of the study on early neonatal and neonatal mortality were 80.20 and 97.23 respectively.

## Discussion

The retrospective cohort study was part of a larger study and was conducted at two hospitals, a tertiary and secondary hospital. A total of 35,649 live newborns participated in the study. Adjusted HR on early neonatal mortality showed respiratory distress was the highest risk, followed by asphyxia, maternal mortality, extra-health facility, symmetrical SGA, preterm-AGA, LBW, primary health facility and boys consecutively. Similar result was found in the neonatal mortality.

Early neonatal and neonatal mortality in 4 categories by the survival analysis revealed the highest CMI in preterm SGA, preterm-AGA, and full-term SGA, respectively. Meanwhile, the lowest was found in full-term-AGA. Analysis of 5-year period unveiled the highest CMI in preterm-SGA during 1998–2002. The highest CMI based on the four categories, however, was found in preterm-SGA.

Our study showed 1.2% preterm-SGA and 6.4% full term-SGA, which were similar to the results by Kristensen et al. [27]. However, they were different from Katz et al., which showed 3,4% preterm-SGA and 20,26% full-term SGA by using Alexander’s curve from the USA, for which the 1996 data were based on National Centre of USA. Prevalence of SGA will decrease if a local population reference or a reference from low-middle countries is used [10].

Early neonatal death and neonatal death in our study were higher than Lee’s result whose neonatal death was 15.9/1000 live births. We used the hospital-based data, whereas Lee used the population-based data [2]. Hospital-based study is different from the population-based study because it is defined secondarily to the identification of cases. Cases are selected regardless of the population from which they arise (e.g. all cases from a given hospital receive patients from different settings). Also, hospital - based study included the infants with morbidity, resulting in more cases of death. Population-based study, on the other hand, refers to a defined population (e.g., a study, a project); as opposed to based on a hospital, or by contrast with programs or studies with no population reference, frame, or base. Pertaining to a general population defined by geopolitical boundaries; this population is the denominator and/or the sampling frame [28]. In general, the neonatal condition in Sardjito hospital was more severe than that in Sleman hospital.

Generally, pre-term SGA infants are more likely at risk of mortality due to the two challenges preterm and SGA, than preterm AGA. In our study, however, if maternal and other neonatal problems were included as confounding variables, preterm-AGA would seem to display higher and more significant Hazard Ratio. Meanwhile, when survival analysis on the four groups of preterm/full-term and SGA/AGA was conducted, the highest CMI was identified in Preterm-SGA for early neonatal mortality, neonatal mortality, and 5-year-period of neonatal mortality. Katz et al., reported that the highest risk of neonatal mortality by gestational age and AGA-SGA was preterm SGA, with full-term AGA as a reference [4, 9]. Preterm SGA may be associated with increased risk of neonatal mortality [11, 27, 29–32]. Grisaru-Granovsky reported that the mortality risk of SGA infants would increase if their birth weight was < 3 percentile [32]. Risks of stillbirth and neonatal death were two to three times higher in preterm SGA in low and medium income countries, except in the very high human development index. Full-term-SGA was significantly associated with perinatal death irrespective of the categories [33].

Our study showed the symmetrical SGA had higher HR on early and neonatal mortality than did asymmetrical SGA. The symmetrical SGA group showed a consistently higher risk of death during the neonatal period [34, 35] The early neonatal mortality of symmetrical SGA infants with short crown-heel length was significantly greater than that of asymmetrical SGA infants with normal crown-heel length. However, a slightly but not significantly greater mortality was observed in asymmetrical SGA versus symmetrical SGA using Ponderal Index regardless of the method of SGA used [36]. Symmetrical SGA infants increased the risk of chronic lung disease, needed longer hospital stay, and had higher consumption of oxygen and continuous positive airway pressure than did asymmetrical SGA [35].

Asphyxia in our study affected early neonatal mortality with HR 5,08 and neonatal mortality with HR 4,32. Another study reported that birth asphyxia deaths accounted for 30% of neonatal mortality [37] Globally, the main direct causes of neonatal death are estimated to be preterm (28%), severe infections (26%), and asphyxia (23%) [38]. Indonesia Ministry of Health noted that asphyxia was responsible for 27% of neonatal mortality [18].

In our study, respiratory distress on adjusted HR revealed the highest HR on early neonatal mortality and neonatal mortality. Sharma found there was no change of risk in respiratory distress syndrome in SGA infants at gestational age ≤32 weeks, but the risk for it significantly decreased at gestational age >32 weeks. Some studies confirm preterm SGA have higher risk of developing chronic lung disease [11, 29–32] Respiratory distress in the preterm SGA maybe associated with increased risk of utilization [30]. In the cohort of late preterm birth, there was no significant difference in the rate of composite respiratory morbidity between SGA and AGA neonates [39]. Another study reported that complications of respiratory distress in SGA neonates prior to hospital discharge included length of hospital stay, highest use of mechanical ventilator, and neonatal mortality with the highest CMI in preterm SGA [49].

In this study, LBW demonstrated a higher HR on early and neonatal mortality. Low birth weight infants were about 2–10 times more likely to die than were heavier infants. Despite substantial progress over the last 10 years, the survival, health, growth, and neurodevelopment of preterm and LBW infants remained a concern in many countries. The reasons included the complexities of caring for these vulnerable infants and preventing complications [40].

Since sepsis was insignificant by adjusted HR analysis, it was not categorized into early and late. The SGA neonates were not at an increased risk for early onset of neonatal sepsis [41, 42]. While early onset of sepsis in the two groups did not pose differences, late onset of sepsis increased the risk of neonatal mortality [42].

Our study also revealed that boys had HR 1,16 on early neonatal mortality and HR 1,12 on neonatal mortality. This was similar to the result of Tamayev’s study which reported boys and placental fetal inflammatory responses, and villitis of unknown etiology are independently associated with adverse neonatal outcome in SGA neonates [43].

Mother’s mortality demonstrated HR 2,27 on early neonatal death and HR 2,46 on neonatal death. Experience of maternal death may likely cause mothers to have infants with greater risks of mortality, and the survival trajectory of these children is far worse than that of mothers who do not die postpartum [44]. Another study reported that infants whose mothers died during delivery or shortly after were up to 7 times more likely to die within the first month of life than those whose mothers survived [45]. This highlights the importance of investigating how clinical care and socio-economic support programs can better address the need of orphans, both throughout the intra and postpartum periods, as well as over the life course [44].

Increased HR neonatal mortality through delivery at first health facility was 1,31 and the same risk through delivery outside health facility increased by 1,97. Similar result was found in early neonatal death. Another study revealed that infants born outside the hospital had a mortality risk with Odds ratio 1,7 (IK 95% 1,2–2,5) even though it had been adjusted to the perinatal risk and severity of the disease [46]. The result was consistent with the importance of encouraging tertiary hospital delivery for high risk infants [46, 47]. Other studies demonstrated that preterm infants born in villages had a higher risk of neonatal mortality rate than those delivered in urban areas [48] We therefore suggest that high risk antenatal fetus be referred to a tertiary hospital regionalization in order to reduce the possible harmful risks.

Decreased neonatal mortality during the 5-year-period suggested improvement of neonatal care in the health facilities despite the fact that SDG target had not been met. Improved quality of health providers, emergency equipment, and the development of neonatal intensive care units every 5 years exhibited decreased CMI. Meanwhile, the highest CMI was found in preterm SGA infants.

The use of cohort retrospective was the weakness of our study, since it had potentials to miss some data. The study consisted of only one tertiary hospital and one secondary district hospital due to limited funds and operational problems. The high number of sample was the strength of our study. Investigation was carried out for 20 years in order to analyse and compare the mortality every 5 years.

## Conclusion

Respiratory distress was the highest HR in early and neonatal mortality. Survival analysis showed the highest CMI on early and neonatal mortality was identified in preterm-SGA, full-term-SGA, and full-term-SGA, respectively. The 5 - year - period of neonatal mortality revealed the highest CMI during 1998–2002 period, whereas based on 4 SGA categories, preterm-SGA demonstrated the highest CMI.

This information may help future research in developing strategies that may contribute to the reduction of neonatal mortality, such as improvements in health care for more susceptible newborns like SGA and/or preterm infants.

## Data Availability

The data are stored in the Child Health Department, Faculty of Medicine, Public Health and Nursing, Universitas Gadjah Mada. Those who are in need of the data can contact the corresponding author.

## Abbreviations

AGA: Appropriate gestational age
CI: Confident interval
CMI: Cumulative mortality incident
HR: Hazard ratio
MP: Maternal Perinatal
LBW: Low birth weight
SEAR NPD WHO: South East Asia Region Neonatal-Perinatal Data World Health Organization
SGA: Small for gestational age
